# Measuring and explaining multi-directional inefficiency in the Malaysian dairy industry

**DOI:** 10.1108/BFJ-11-2016-0549

**Published:** 2017-12-04

**Authors:** Nurul Aisyah Binti Mohd Suhaimi, Yann de Mey, Alfons Oude Lansink

**Affiliations:** 1 Chair Group Business Economics, Wageningen University and Research, Wageningen, The Netherlands; 2 Fakulti Biosumber dan Industri Makanan, Universiti Sultan Zainal Abidin, Kampus Tembila, Terengganu, Malaysia

**Keywords:** Malaysia, Dairy industry, Multi-directional efficiency analysis, Technical inefficiency

## Abstract

**Purpose:**

The purpose of this paper is to measure the technical inefficiency of dairy farms and subsequently investigate the factors affecting technical inefficiency in the Malaysian dairy industry.

**Design/methodology/approach:**

This study uses multi-directional efficiency analysis to measure the technical inefficiency scores on a sample of 200 farm observations and single-bootstrap truncated regression model to define factors affecting technical inefficiency.

**Findings:**

Managerial and program inefficiency scores are presented for intensive and semi-intensive production systems. The results reveal marked differences in the inefficiency scores across inputs and between production systems.

**Practical implications:**

Intensive systems generally have lowest managerial and program inefficiency scores in the Malaysian dairy farming sector. Policy makers could use this information to advise dairy farmers to convert their farming system to the intensive system.

**Social implications:**

The results suggest that the Malaysian Government should redefine its policy for providing farm finance and should target young farmers when designing training and extension programs in order to improve the performance of the dairy sector.

**Originality/value:**

The existing literature on Southeast Asian dairy farming has neither focused on investigating input-specific efficiency nor on comparing managerial and program efficiency. This paper aims to fill this gap.

## Introduction

The demand for dairy products in the Asian region (including Malaysia) has doubled over the past decade. Currently, Malaysia still relies heavily on imports to satisfy its domestic demand for dairy products. Although milk production increased over the past decade, the growth was insufficient to meet the growing domestic demand for fresh milk. In 2012, domestic production accounted for only 14.06 percent of total consumption (DVS, 2012). Even though Malaysia does not have a comparative advantage in dairy production, the government uses tariffs to protect domestic markets and there is no export of dairy products to international markets (Peng and Cox, 2006). Recently, the dairy sector was selected by the Malaysian Government as an Entry Point Project (EPP) under the National Key Economics Area program. The EPP aims among other things to reduce Malaysia’s dependence on imported fresh milk in order to increase food security by forming dairy clusters under anchor companies to produce milk on a large-scale basis. The dairy industry can increase its production among others by improving the technical efficiency of the use of inputs such as land, feed, and labor. It remains a question though how dairy farms can improve their technical efficiency. Moreover, what factors determine the technical efficiency and for what inputs specifically can savings be obtained?

Dairy farming in Malaysia is practiced in two main types of production systems: the intensive and semi-intensive system (note that in practice, some farms might hinge on the borderline between them of course). In the intensive system, grazing animals are confined to a small area on which no feed is produced and the animals are fed on stored feed. Farmers feed their cattle following a schedule. In the semi-intensive system, animal graze on land that is also used for crop production. Ruminants, such as buffalo, cattle, and goats, are free to move under crop production, such as palm oil and rubber estate. This type of system uses less concentrated feed and labor, but requires more land than the intensive system. Intensive farms are more likely to have higher operational costs compared to semi-intensive farms. Hence, an analysis of technical efficiency of the Malaysian dairy industry should distinguish technical efficiency given the system under which the farm operates (intensive vs semi-intensive) from efficiency differences between the two systems. In what follows, this paper refers to the efficiency within a system as managerial efficiency whereas differences in efficiency between systems are referred to as program efficiency. This approach of program (and managerial) efficiency was conceived by Charnes *et al.* (1981). Agricultural program efficiency has been considered by, for example, Gómez-Limón *et al.* (2012), who estimated the program efficiency of traditional rain-fed mountain groves, traditional rain-fed plain groves, and irrigated intensive groves. Using the same approach, Beltrán-Esteve and Reig-Martínez (2014) assessed conventional and organic citrus grower efficiency in Spain.

According to Koopmans (1951), a producer is technically efficient if output can only be increased when at least one other output is reduced or at least one input is increased, or if a reduction in any input requires an increase in at least one other input or a reduction in at least one output. Producers directly benefit from improvements in input usage because more efficient farms tend to generate a higher income and have a better chance of staying in business (Bravo-Ureta and Rieger, 1991; Dartt *et al.*, 1999; Lawson *et al.*, 2004). Non-parametric methods, such as data envelopment analysis (DEA), calculate the individual efficiency scores of decision-making units – such as dairy farms – by relating each farm’s performance to a benchmark of the best practice farms (Tauer, 1993; Weersink *et al.*, 1990). This paper uses a multi-directional efficiency analysis (MEA) instead, as this enables us to investigate in greater detail potential differences in input utilization. By calculating both managerial and program input-specific efficiency scores, this approach allows us to present a detailed overall idea about the differences among inputs and between farming systems. There are studies focusing on the technical efficiency of farms in Malaysia, for example, Serin *et al.* (2008) identified the efficiency of the resources used in the beef cattle production in Johor and Iinuma *et al.* (1999) estimated the technical inefficiency of carp pond culture in Peninsula Malaysia. The existing literature on Malaysian dairy farming, however, has not investigated the efficiency of the use of inputs. Also, no study compared the managerial and program efficiency of Malaysian dairy farms. Performing such an analysis would provide valuable information to policy makers and business actors that aim at decreasing the dependence of Malaysia on dairy imports.

Therefore, the objectives of this study are: to estimate the input-specific technical inefficiency of Malaysian dairy farms in terms of both managerial and program inefficiency and to identify the factors affecting the technical inefficiency scores. For the first step, the paper uses an MEA. For the second step, the managerial MEA inefficiency scores are regressed on potential determinants using a single-bootstrap truncated regression model. This is the first paper to analyze the inefficiency of dairy farming in Malaysia, using an MEA framework and a single-bootstrap truncated regression approach to explain observed differences in managerial inefficiency. Scrutinizing the role of technical inefficiency in Malaysian dairy production can serve as an example to other Asian countries, especially in Southeast Asia. First, they have a similar climate which is tropical-hot and humid all year round with plentiful rainfall. Second, most of the dairy herds in Asia are owned by smallholders. Finally, in most of the Asian tropics, cattle production systems are also primarily grass based with cows either allowed to graze freely or confined and provided with cut-and-carry harvested forages (Herath and Mohammad, 2009).

## Materials and methods

This research adopts a two-stage approach. First, we employ MEA to measure technical inefficiency for specific inputs used in the production of milk on dairy farms in Malaysia. Second, a single-bootstrap truncated regression model is used to explain the determinants of technical inefficiency in Malaysian dairy farming. As there are two distinct production systems in our sample, we run the regression analysis separately for each system.

### Multi-directional efficiency analysis

Following Bogetoft and Hougaard (1999) and Asmild *et al.* (2003), we identify a set of *k*=1, …, *K* farm observations. Each farm uses *N* inputs, *x*=(*x*_1_, …, *x*_*N*_) and produces one output, *y* (total revenue). We assume a constant technology of production and that all farmers produce a homogenous product. In input-oriented MEA, an ideal point (x^*^, *y*^0^) for the farm under analysis (*x*^0^, *y*^0^) is first identified by considering sub-vector efficiencies for each dimension of the inputs separately, i.e. by solving five linear programming problems for *i*=1, …, *N* as follows:

$$x_{i}^{*}=\min_{x_{i},\;\lambda^{k}}x_{i}$$

s.t.:

(1) $$\begin{array}{l} \sum_{k=1}^{K}\lambda^{k}x_{i}^{k}⩽x_{i}\\ \sum_{k=1}^{K}\lambda^{k}x_{-i}^{k}⩽x_{-i}^{0}\;\\ \sum_{k=1}^{K}\lambda^{k}y^{k}⩾y^{0}\\ \sum_{k=1}^{K}\lambda^{k}=1\\ \lambda^{k}⩾0,\;\;k=1,\;2,\;\ldots ,\;K, \end{array}$$

where $\sum_{k=1}^{K}\lambda^{k}=1$ imposes variable returns to scale. Solving Equation (1) for each input provides the input coordinates of the ideal point, $x^{*}=\left(x_{1}^{0},\;x_{2}^{*},\;x_{3}^{*},\;x_{4}^{*},\;x_{5}^{*}\right)$. Note that *x*^0^=x^*^ implies that *x*^0^ is an efficient farm. Unlike DEA, where input adjustments are made in proportion to the input mix, MEA considers adjustments in proportion to the improvement potentials (*x*^0^−x^*^) (Asmild *et al.*, 2016). Thus, a vector of input-specific efficiencies is found by solving the following linear programming problem:

$$\beta^{*}=\max_{\beta ,\lambda^{k}}\beta$$

s.t.:

(2) $$\begin{array}{l} \sum_{k=1}^{K}\lambda^{k}x_{i}^{k}⩽x_{i}^{0}-\beta \left(x_{i}^{0}-x_{i}^{*}\right),\;i=1,\;2,\;3,\;4,\;5\\ \sum_{k=1}^{K}\lambda^{k}y^{k}⩾y^{0}\\ \sum_{k=1}^{K}\lambda^{k}=1\\ \lambda^{k}⩾0,\;k=1,\;2,\;\ldots ,\;K \end{array}$$

and input-specific MEA inefficiency scores for farm (*x*^0^, *y*^0^) are calculated as:

(3) $$ie_{i}=\frac{\beta^{*}\left(x_{i}^{0}-x_{i}^{*}\right)}{x_{i}^{0}},\;i=1,\;2,\;3,\;4,\;5$$

The inefficiency scores (*ie*_*i*_) take values between 0 and 1, where a value of 0 indicates no improvement potential on the variable in question when a firm is efficient, and 1 otherwise.

**Figure 1:**
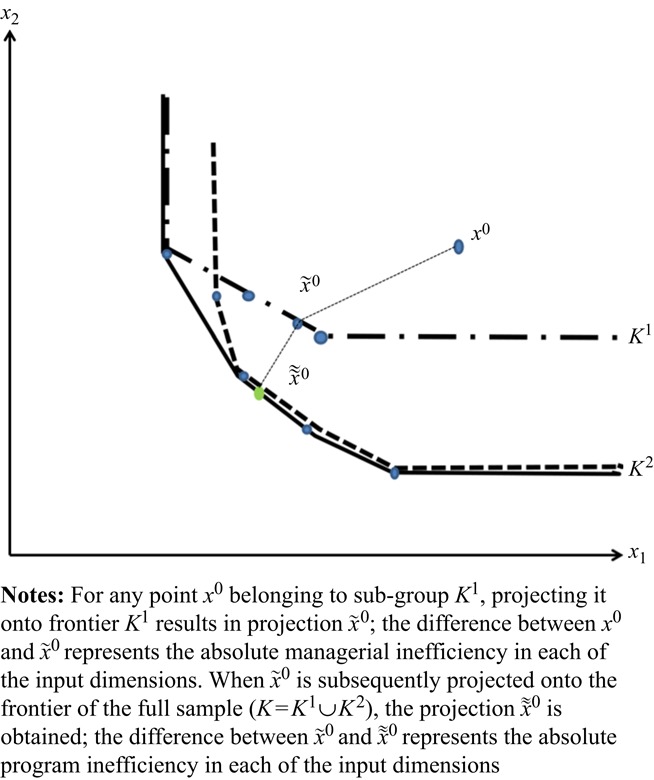
Multi-directional efficiency analysis assessment of managerial and program inefficiency for two sub-groups *K*^1^ and *K*^2^

We follow Asmild *et al.* (2016), by using the MEA approach to estimate managerial and program inefficiency. The MEA managerial inefficiency scores are found by applying Equations (1) and (2) to each sub-sample of intensive and semi-intensive farms. Then, we replace the observations by their sub-sample-specific MEA benchmarks, ${\tilde{x}}_{i}^{0}=\left(1-ie_{i}x_{i}^{0}\right)$ for all *i* to obtain a new set of observations. Running Equations (1) and (2) for this new set of observations provides the program inefficiency scores. Figure 1 illustrates the concept of MEA managerial and program inefficiency for two sub-groups (*K*^1^ and *K*^2^). In Figure 1, *x*^0^ in *K*^1^ is first projected onto frontier *K*^1^, in the direction of the MEA ideal point, resulting in projection ${\tilde{x}}^{0}$. The difference between *x*^0^ and ${\tilde{x}}^{0}$ is the absolute managerial inefficiency in each of the input dimensions. ${\tilde{x}}^{0}$ is subsequently projected onto the frontier of the full sample, *K*=*K*^1^∪*K*^2^, resulting in the projection ${\tilde{\tilde{x}}}^{0}$, and the difference between ${\tilde{x}}^{0}$ and ${\tilde{\tilde{x}}}^{0}$ is the absolute program inefficiency in the input dimension.

### Single-bootstrap truncated regression model

The single-bootstrap truncated regression method, developed by Simar and Wilson (2007), is used for the second stage of the analysis. Estimated DEA efficiency scores are serially correlated (Simar and Wilson, 2007; Xue and Harker, 1999) and hence using these scores in a standard ordinary least squares regression analysis results in a violation of the basic assumption of independence within the sample values (Simar and Wilson, 2011). Assuming that MEA scores are also serially correlated, we use a single-bootstrap regression model with left truncation to determine the factors affecting managerial inefficiency. The model for the single-bootstrap truncated regression is as follows:

$${\hat{\delta }}_{i}=Z_{i}\beta +\varepsilon_{i}$$

where the dependent variable ${\hat{\delta }}_{i}$ is the estimated technical inefficiency score, *Z* is a vector of independent variables, *β* its associated vector of coefficients, and *ε*_*i*_ the idiosyncratic error term. The intensive and semi-intensive systems have different management practices, thus we assume that the independent variables may affect inefficiency differently in each system. Hence, we run the single-bootstrap truncated regression separately for each system.

According to Simar and Wilson (2007), the confidence intervals for the coefficients of the second-stage regression, which are appropriate for inference, can be constructed as follows (Algorithm I):

1. Perform the MEA approach to get inefficiency score, ${\hat{\delta }}_{i}$, for each firm *i*=1, …, *n*.
2. Regress ${\hat{\delta }}_{i}$ on the independent variables, *Z*_*i*_, using left truncation at 0 (i.e. only the inefficient observations are included) to obtain estimates $\hat{\beta }$ and ${\hat{\sigma }}_{\varepsilon }$ of the parameters *β* and *σ*_*ε*_.
3. Repeat the following three steps below *B* (1,000 bootstrap iterations) times to obtain a set of bootstrap estimates $B^{*}={\left\{\left({\hat{\beta }}^{*},\;{\hat{\sigma }}_{\varepsilon }^{*}\right)\right\}}_{b=1}^{B}$:
  - For each *i*=1, …, *n*, draw $\varepsilon_{i}^{*}$ from the $N\left(0,\;{\hat{\sigma }}_{\varepsilon }^{2}\right)$ distribution with left truncation at $\left(0-Z\hat{\beta }\right)$.
  - For each *i*=1, …, *n*, compute $\delta_{i}^{*}=Z_{i}\hat{\beta }+\;\varepsilon_{i}^{*}$.
  - Regress $\delta_{i}^{*}$ on the independent variables, *Z*_*i*_, using left truncation at 0 to obtain ${\hat{\beta }}_{b}^{*}$ and ${\hat{\sigma }}_{\varepsilon ,b}^{*}$.
4. Obtain the mean and 95% confidence interval of the *β*s and *σ*.

## Data description

We collected original data from Malaysian dairy farms using a two-stage stratified sampling design. First, we purposely selected four distinct production regions in Malaysia based on the most representative milk production: Johor (43), Negeri Sembilan (54), Selangor (42), and Melaka (61). Within each region, respondents were then chosen using two types of sampling: convenience sampling and random sampling[1]. For the convenience sampling, we waited for the farmers who were going to sell their milk to the PPIT. In order to reach our target sample of 200 respondents, we then turned to random sampling by randomly choosing dairy farmers from the complete list of dairy farms which was provided to us by the Department of Veterinary Services. Personal interviews were conducted among these owners or managers of dairy farms between February and June 2015. The questionnaire includes the use of dairy inputs and outputs, farm revenue, the material and equipment used for farming, socio-economic factors, farm characteristics, and transaction cost variables. Our final sample consists of 200 Malaysian dairy farms, classified into the intensive (*n*=100) and semi-intensive (*n*=100) systems. Our data are one-time cross-sectional data which reflect the activities of farmers in the production year 2014.

### Data for the MEA

For the MEA, we consider one output and five inputs. Summary statistics for these variables are shown in Table I.

Output is total revenue calculated as the sum of annual sales of milk and cattle, other sales, and own consumption. The first two components are estimates provided by the farmers in the local currency Ringgit Malaysia (MYR). Own consumption, however, is measured as the product of average consumption per capita (36.89 liter in 2007, Food and Agriculture Organization), the number of family members, and the average selling price in the sample for milk sold to the state-owned enterprise, Dairy Industry Service Centre, and milk sold directly to consumers. The inputs are land, labor, herd size, feed, and other expenditure.

Land size is measured as the number of hectares used by the farmer for farming activities. Land also includes land rented for dairy activities. The land size ranged from 0.1 ha to 323.7 ha. The large variation in land size is due to the differences between intensive and semi-intensive systems.

Labor is defined as the total labor used for dairy activities, including family and hired labor but excluding the farm operator, measured in number of persons. Labor ranged from 0.1 to 12 persons.

Herd size is defined as the number of cows that a farmer owned and it is measured in tropical livestock units. Using tropical livestock units, we assumed that 1 calf is equivalent to 0.2 cow.

Feed is defined as the total cost of purchased feed for cattle and measured in MYR. The total value was obtained by asking farmers how much they spent annually on a few types of feed typically used for dairy farming in Malaysia (including an option “other”) and then adding all components.

Other expenditures (in MYR) are defined as expenditures on other goods and services, which include farmers’ estimates of breeding expenses, veterinary services and medicines, farm maintenance, and other expenses.

### Data for the single-bootstrap truncated regression model

The existing literature suggests that farming efficiency might be affected by variables such as age of the farmer (Binam *et al.*, 2003; Heriqbaldi *et al.*, 2014), years of experience (Tzouvelekas *et al.*, 2002; Amaza and Olayemi, 2002), family size (Binam *et al.*, 2003), off-farm employment (Wang *et al.*, 2013), access to credit (Mlote *et al.*, 2013), and availability of extension services (Mutai *et al.*, 2013). The following paragraphs discuss each determinant considered in this study and its measurement in more detail. Table II presents summary statistics of the independent variables for our sample.

Farmer’s age may have a positive relation with technical inefficiency. Older farmers may not be up to date with new technology, machinery, and equipment, and may have less energy to conduct farm activities. For example, Coelli *et al.* (2002) found that younger rice farmers in Bangladesh were more efficient than older rice farmers. In this study, farmer’s age is measured as the age of the farmer in the year 2015.

Experience in dairy farming is expected to negatively affect inefficiency as it can be considered as informal training for farmers. Thus, an increase in experience is assumed to decrease the technical inefficiency of dairy farming. Singbo and Oude Lansink (2010) showed that technical inefficiency was negatively affected by the number of years of experience in lowland farming in Benin. Gelan and Muriithi (2012) found that experience had a positive effect on the efficiency of dairy farms in East Africa. Experience is measured as the number of years the farm operator has been operating the dairy farm.

Hallam and Machado (1996) argued that there is little evidence that higher levels of facilities, machinery, or equipment (such as milking parlors and free-stall housing) are associated with increased efficiency. However, Filipovic and Kokaj (2009) found that using a milking machine instead of hand milking can increase work efficiency on small family farms in Croatia. Thus, having a larger number of portable milking machines is expected to decrease the technical inefficiency of dairy farms. We measured this variable as the number of portable milking machines available to farmers.

At the start of the development of the Malaysian dairy industry, the sector was heavily subsidized by government (Wells, 1981). However, in recent years, the government has gradually reduced subsidies to limit government dependency. Erjavec *et al.* (2003) showed that subsidies that are provided as a supplement to farm income can – as an unintended consequence – increase the level of technical inefficiency, as farmers might reduce their efforts. Similarly, Bojnec and Fertő (2013) showed that subsidies negatively impact farm technical efficiency, as acquiring subsidies makes the farmer less motivated. In this study, government finance is thus expected to have a positive influence on technical inefficiency. This variable is measured as the proportion of finance received from the government in total revenue (including the finance received from the government). Introducing subsidies in this way prevents any potential multicollinearity with the number of portable milking machines and allows for easy interpretation of its coefficient.

## Results and discussion

### Managerial efficiency analysis results

Using the General Algebraic Modeling System (2013) software package, MEA was applied to each sub-sample to determine the managerial inefficiency score for the intensive and semi-intensive systems. The mean and median scores of MEA input-specific managerial inefficiency are provided in Table III. As the distributions for all inputs are negatively skewed, we also provide the median values, which may give a better representation of central tendency than the mean. Intensive farms, on average, have input-specific managerial inefficiency scores of 0.590, 0.555, 0.499, 0.513, and 0.545 for land, labor, herd size, feed, and other expenditure, respectively. These results suggest that the intensive farms in our sample can reduce the use of land by 59 percent, labor by 56 percent, herd size by 50 percent, feed costs by 51 percent, and other expenditure by 55 percent and still produce the same level of revenue. The semi-intensive farms in our sample, on average, can reduce land by 62 percent, labor by 44 percent, herd size by 51 percent, feed by 57 percent, and other expenditure by 54 percent and still produce the same level of revenue.

This finding indicates the intensive farms are more managerially efficient than the semi-intensive farms for all inputs, except labor and other expenditure. For the intensive farms, land is the most inefficient input, followed by labor and other expenditures. Herd size has the lowest score for managerial inefficiency as expected because this system keeps animals in the shed, which makes it more convenient for a farmer to manage more animals. For the semi-intensive system, land has the highest score for managerial inefficiency, followed by feed, other expenditure, and herd size. Labor has the lowest score for managerial inefficiency for the semi-intensive system.

Overall, the MEA results show that the Malaysian dairy farms in both systems are technically inefficient in their use of inputs. This indicates that substantial amounts of input can be saved while maintaining the current level of output. Technical inefficiency of land is high for both systems. This can be explained by the routines of the farmers, as the farmers who have land are hesitant to use it for other activities such as planting a grass or other crop. Most farmers do not grow their own pasture. They tend to purchase feed or obtain it from abandoned land. Some of the farmers operate a large land area, especially in the semi-intensive system, and should consider having their own pasture area to maximize their land usage. For the intensive system, on average, labor has the second-highest inefficiency level, whereas labor has the lowest inefficiency level for the semi-intensive system. This was expected because the intensive system is more labor-intensive than the semi-intensive system. This implies that intensive farms can reduce labor by 56 percent and still produce the same output. Therefore, farmers could allocate this labor to other productive activities. Feed has the second-highest inefficiency in the semi-intensive system. This result implies that farmers can reduce feed by 63 percent and still produce the same output. This was expected, as farmers purchase large amounts of feed even though cattle are allowed to graze by themselves. This result suggests the farmers can limit their purchases of feed by better monitoring the amount of daily feed needed in order to better predict the total amount of feed required. The results also show that only nine farms in the intensive system and six farms in the semi-intensive are efficient.

**Figure 2:**
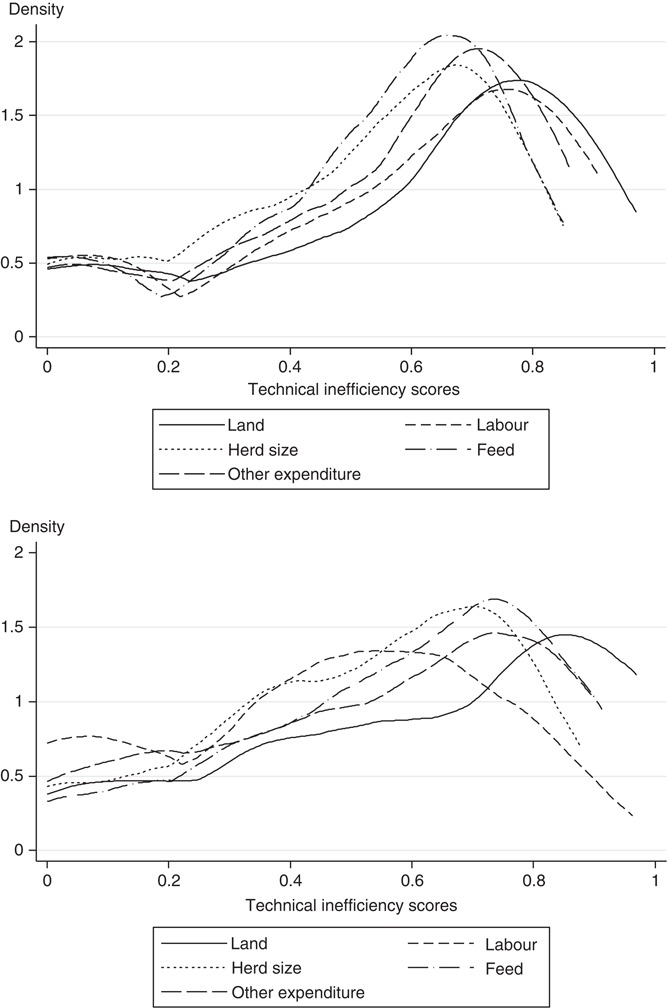
Kernel density plots of managerial inefficiency scores in the intensive (upper panel) and semi-intensive (lower panel) systems

Figure 2 shows the distribution of managerial inefficiency scores for the intensive and semi-intensive systems. The upper panel of Figure 2 shows that the patterns of inefficiency scores are quite similar across the different inputs in the intensive system. Farmers are mostly clustered at the 0.7 inefficiency level, especially for feed. The distribution of farmers is flatter in the middle classes (0.1 to 0.3). At the same time, there is a cluster of the most-efficient farmers at the 0 technical inefficiency level. The differences between average and median values in both systems suggest that distributions of inefficiency for all inputs are negatively skewed. The lower panel of Figure 2 shows that the patterns of inefficiency scores are less similar across inputs in the semi-intensive system. There is clustering of the most-efficient farmers at the 0 technical inefficiency level, especially for labor. The distribution of technical inefficiency scores is flatter (platykurtic) for labor compared to the other inputs. The difference in the distributions of technical inefficiency indicates that farmers perform differently in managing their inputs in the intensive and semi-intensive systems.

### Program efficiency analysis results

The average program inefficiencies for the intensive and semi-intensive systems are shown in Table III. The program efficiency can be assessed by comparing managerial efficient units to the frontier spanned by both farm types. The program inefficiency scores for the intensive systems are very close to 0. Accordingly, the frontier for the intensive system is almost identical to the pooled frontier. This implies that the intensive system can be considered best practice in general. By considering program inefficiency, MEA shows that there are significant differences not only between farm types, but also between inputs. Across these two farm types, the highest – both managerial and program – inefficiency is on land, which suggests that farmers generally have enough land available to them but do not optimize the use of land. Intensive farms, on average, have lower program inefficiency scores than semi-intensive farms. This means that intensive farms perform better than semi-intensive farms. As the intensive system also has the lowest managerial inefficiency compared to the semi-intensive system, we conclude that the intensive system is the best-performing farm system. This may be due to differences in the mode of production, quality of feed, and breed of cattle. For the intensive system, the program inefficiency score of labor is the lowest, whereas the managerial inefficiency scores of labor are the second highest. For the semi-intensive system, land has the highest score for program inefficiency and other expenditure has the lowest program inefficiency score.

As the intensive system is already the preferred farming type in Malaysian dairy farming – 70 percent of dairy farmers run their farm using this system – there is some, albeit limited, scope for specific policies aimed at encouraging farmers to move from the semi-intensive to the intensive system. In addition, our results suggest that the program efficiency of the semi-intensive system could be improved as well, for example, through research on novel production technologies tailored to the semi-intensive setting and targeted training on farming activities.

### Single-bootstrap truncated regression results

A single-bootstrap truncated regression model was estimated using the Stata software package (StataCorp., 2011). The results in Tables IV and V show the factors affecting the input-specific managerial technical inefficiency of dairy farming in Malaysia, for the intensive and semi-intensive systems. For the intensive system (Table IV), the number of portable milking machines has a negative relation with the inefficiency of land and herd size. This result implies that the number of portable milking machine units in dairy farming makes farmers more efficient in the use land and herd. This result is in line with a previous study by Castro *et al.* (2012), who found that use of an automatic milking machine (in this paper we refer to a portable milking machine) can increase milk production in Galicia, Spain. However, Steeneveld *et al.* (2012) found that an automatic milking machine did not affect the efficiency of Dutch dairy farms. The age of farmers has a positive relation with the technical inefficiency of labor. This result indicates that older farmers are *ceteris paribus* more inefficient. This result is in line with the study by Lachaal *et al.* (2002) in Tunisian dairy production, indicating that older farmers who lack motivation are less efficient. However, the result is inconsistent with the study by Zhengfei and Oude Lansink (2006) for Dutch agriculture. Experience has a negative relation with the technical inefficiency of other expenditure. This result indicates that more experience decreases the technical inefficiency of a farmer in managing other expenditures. This is expected, because experience helps the farmers to better estimate the cost of other expenditures.

For the semi-intensive system (Table V), the number of portable milking machines has a negative relation with land and other expenditures. The coefficient of portable milking machines suggests that having more portable milking machines can reduce the technical inefficiency of land and other expenditure. Government finance has a positive association with technical inefficiency for feed. This result means that the greater the proportion of finance coming from government support, the greater the technical inefficiency of managing feed. As finance from the government is not only specific for feed, farmers can use it for other farming activities and this could result in a lower efficiency in managing feed. This result is in line with Karagiannis and Sarris (2002) for wheat and mixed arable crop in Greece, Zhu and Oude Lansink (2010) for German, Dutch, and Swedish crops farms, and Iraizoz *et al.* (2005) for Spanish livestock farms.

## Conclusions

The objectives of this study were to investigate the technical inefficiency, decomposed into managerial and program inefficiencies, of dairy farming in Malaysia, and to identify the sources of managerial inefficiency. MEA was used to estimate technical inefficiency for individual inputs under variable returns to scale. The results for managerial inefficiency suggest that intensive farms can maintain their current production level and save 59 percent of land, 56 percent of labor, 50 percent of herd size, 51 percent of feed, and 55 percent of other expenditures. Semi-intensive farms, on average, can save 62 percent of land, 44 percent of labor, 51 percent of herd size, 57 percent of feed, and 54 percent of other expenditures and still produce the same level of output. The application of the MEA approach shows that there are substantial input-specific production inefficiencies among farms for both systems and these dairy farms could increase their production through the improvement of technical efficiency. These results show that valuable insight can be gained from the input-specific inefficiency scores, which are obtained using MEA, which could help farmers to identify which inputs were overused and hence should be reduced.

Our program efficiency MEA results furthermore show that there are significant differences not only in the levels of inefficiencies of the different inputs, but also between the two main farming types in Malaysia. Of the 100 dairy farms sampled for each system, only 9 percent of intensive farms and 6 percent of semi-intensive farms were fully efficient. Based on the percentage of fully efficient farms, farms are similar in efficiency between the two systems. Semi-intensive farms have higher inefficiency scores for all inputs except labor and other expenditure. Semi-intensive farms also have higher program inefficiency scores for all inputs. We therefore conclude that semi-intensive farms are managerially inefficient (except for labor and other expenditure; labor has the lowest inefficiency score among inputs for both systems) and also program inefficient. This may be because of different practices between the intensive and semi-intensive systems. The lower program inefficiency of semi-intensive farms suggests that additional efforts are needed to improve its performance, e.g. by additional research and development into improving technologies tailored to this system. Alternatively, policy makers could use this information to improve the efficiency of Malaysian dairy farmers by advising them to convert to the intensive system.

In the second stage of this study, single-bootstrap truncated regression models were used to investigate the factors affecting the input-specific managerial inefficiency scores. The results of the single-bootstrap truncated regression models for intensive system show the following: the number of portable milking machines has a negative relation with technical inefficiency scores of land and other expenditure, age has a positive relation with labor inefficiency, and experience has a negative relation with other expenditure. For the semi-intensive system, the number of portable milking machines has a negative relation with technical inefficiency scores of land and other expenditure and government finance has a positive relation with the technical inefficiency score of feed. Government finance does not appear to improve farm efficiency, especially regarding feed input. This outcome suggests that the government should redefine its policy for providing farm finance. The government could consider providing portable milking machines instead of credit or other subsidies. In this case, extension officers can provide guidance to the farmers on how to use portable milking machines, as these farmers may not be familiar with this technology. Our results further suggest that Malaysian policy makers should target young farmers when designing training and extension programs.

The limitations of this study are that we have a limited number of observations and variables available to explain the differences in technical inefficiency. Future research could use samples stratified to not only get good estimates of input-specific inefficiency scores, but also to maximize observed differences in terms of explanatory variables. Future work could also focus on measuring (input-specific) technical inefficiency of production over time and explore additional explanatory variables that can explain the technical inefficiency scores in the single-bootstrap truncated regression analysis.

## Figures and Tables

**Table I:** Mean and standard deviation of output and inputs used in the multi-directional efficiency analysis (MEA) model

Variable	Unit	Mean	SD
Total revenue	MYR10,000	13.95	11.81
Land	10 ha	7.07	13.71
Labor	Persons	3.09	1.51
Herd size	10 cows	3.15	1.92
Feed	MYR10,000	3.95	4.29
Other expenditure	MYR10,000	1.39	1.26

**Note:** Sample size=200

**Table II:** Mean and standard deviation of variables used in the truncated bootstrap regressions

Variable	Unit	Mean	SD
Age	Years	44.25	11.2
Experience	Years	17.72	10.6
Portable milking machines	Number of machines	1.26	1.17
Government finance	Share of government finance in total farm revenue	0.06	0.09

**Note:** Sample size=200

**Table III:** Mean and median of managerial inefficiency and program inefficiency scores for intensive and semi-intensive systems

	Managerial inefficiency					Program inefficiency				
Farming type	Land	Labor	Herd size	Feed	Other expenditure	Land	Labor	Herd size	Feed	Other expenditure
Intensive										
Mean	0.590	0.555	0.499	0.513	0.545	0.013	0.001	0.011	0.004	0.013
Median	0.713	0.647	0.581	0.597	0.657	0.000	0.000	0.000	0.000	0.000
Semi-intensive										
Mean	0.618	0.441	0.511	0.569	0.540	0.169	0.054	0.083	0.040	0.037
Median	0.696	0.485	0.577	0.635	0.584	0.078	0.000	0.038	0.000	0.000

**Notes:** Sample size=100 for each system. Managerial efficiency scores explain differences in efficiency within one system, whereas differences in efficiency between systems are reflected by the program efficiency scores. The addition of both scores presents the overall differences in inefficiency

**Table IV:** Results of the truncated bootstrap regression model explaining differences in input-specific managerial inefficiency scores of intensive farms

	Land			Labor			Herd size			Feed			Other expenditure		
Variable	Mean	Lower	Upper	Mean	Lower	Upper	Mean	Lower	Upper	Mean	Lower	Upper	Mean	Lower	Upper
Age	0.003	−0.002	0.011	0.008*	0.001	0.014	0.004	−0.002	0.009	0.001	−0.004	0.007	0.004	−0.001	0.010
Experience	−0.006	−0.013	0.002	−0.005	−0.013	0.002	−0.005	−0.012	0.001	−0.002	−0.008	0.005	−0.006*	−0.013	−0.000
Portable milking machines	−0.050*	−0.104	−0.000	−0.013	−0.068	0.039	−0.049*	−0.099	−0.007	−0.031	−0.075	0.013	−0.045	−0.093	0.001
Government finance	0.101	−0.519	0.695	−0.061	−0.737	0.558	0.151	−0.378	0.659	0.241	−0.302	0.791	0.139	−0.424	0.691
Constant	0.617	0.348	0.880	0.356	0.047	0.644	0.518	0.273	0.743	0.552	0.319	0.777	0.564	0.328	0.789
*σ*	0.276	0.233	0.323	0.276	0.234	0.324	0.233	0.199	0.268	0.233	0.197	0.274	0.242	0.203	0.280

**Notes:** Sample size=100. Lower and upper represent the bounds of a 95% confidence interval. Number of truncated observations: 9. We have no indication of the presence of multicollinearity (mean VIF=1.29). *Significant at 5 percent level

**Table V:** Results of the truncated bootstrap regression model explaining differences in input-specific managerial inefficiency scores of semi-intensive farms

	Land			Labor			Herd size			Feed			Other expenditure		
Variables	Mean	Lower	Upper	Mean	Lower	Upper	Mean	Lower	Upper	Mean	Lower	Upper	Mean	Lower	Upper
Age	0.005	−0.002	0.012	−0.003	−0.011	0.003	0.003	−0.002	0.009	0.004	−0.002	0.010	0.002	−0.004	0.009
Experience	−0.005	−0.012	0.003	−0.001	−0.008	0.006	−0.002	−0.008	0.004	−0.002	−0.008	0.004	−0.001	−0.007	0.006
Portable milking machines	−0.070*	−0.143	−0.011	−0.054	−0.133	0.003	−0.035	−0.090	0.010	−0.046	−0.104	0.001	−0.060*	−0.125	−0.004
Government finance	0.662	−0.037	1.375	−0.047	−0.814	0.687	0.562	−0.018	1.113	0.587*	0.009	1.170	0.629	−0.042	1.327
Constant	0.558	0.287	0.805	0.664	0.422	0.911	0.453	0.244	0.657	0.482	0.263	0.697	0.511	0.267	0.760
*σ*	0.285	0.242	0.333	0.265	0.221	0.321	0.230	0.193	0.268	0.237	0.203	0.277	0.262	0.221	0.306

**Notes:** Sample size=100. Lower and upper represent the bounds of a 95% confidence interval. Number of truncated observations: 6. We have no indication of the presence of multicollinearity (mean VIF=1.29). *Significant at 5 percent level

## Note

1. The combination of convenience sampling and random sampling was chosen in favor of full random sampling in order to optimally make use of the time and budget available. The target sample size of 200 respondents was determined in the same vein and is in line with similar program/managerial inefficiency analysis studies (e.g. Gómez-Limón *et al.*, 2012; Beltrán-Esteve and Reig-Martínez, 2014).
